# The impact of levator ani muscle trauma and contraction on recurrence after prolapse surgery

**DOI:** 10.1007/s00192-022-05168-8

**Published:** 2022-03-28

**Authors:** M. Ø. Nyhus, S. Mathew, K. Å. Salvesen, I. Volløyhaug

**Affiliations:** 1 Department of Obstetrics and Gynecology, St. Olavs Hospital, Trondheim University Hospital, Postboks 3250 Torgarden, 7006 Trondheim, Norway; 2 Department of Clinical and Molecular Medicine, Norwegian University of Science and Technology, Trondheim, Norway

**Keywords:** Gynecological surgical procedures, Levator ani muscle, Pelvic floor, Pelvic organ prolapse, Ultrasound imaging

## Abstract

**Introduction and hypothesis:**

The objective was to explore the impact of levator ani muscle (LAM) trauma and pelvic floor contraction on symptoms and anatomy after pelvic organ prolapse (POP) surgery.

**Methods:**

Prospective study including 200 women with symptomatic POP ≥ grade 2 examined 3 months prior to and 6 months after surgery. Prolapse in each compartment was graded using the Pelvic Organ Prolapse Quantification (POP-Q) system, and women answered yes/no to a question about bulge sensation. Pelvic floor muscle contraction was assessed with transperineal ultrasound measuring proportional change in levator hiatal anteroposterior diameter from rest to contraction. LAM trauma was diagnosed using tomographic ultrasound imaging. Statistical analysis was performed using multivariate logistic regression analysis.

**Results:**

A total of 183 women (92%) completed the study. Anatomical recurrence (POP ≥ grade 2) was found in 76 women (42%), and a bulge sensation was reported by 35 (19%). Ninety-two women (50%) had LAM trauma, and this was associated with increased risk of anatomical recurrence (OR 2.1 (95% CI 1.1–4.1), *p* = 0.022), but not bulge sensation (OR 1.1 (95% CI 0.5–2.4), *p* = 0.809). We found a reduced risk of bulge sensation for women with absent to weak contraction compared with normal to strong contraction (OR 0.4 (95% CI 0.1–0.9), *p* = 0.031), but no difference in risk for POP ≥ 2 after surgery (OR 1.5 (95% CI 0.8–2.9), *p* = 0.223).

**Conclusions:**

Levator ani muscle trauma was associated with increased risk of anatomical failure 6 months after POP surgery. Absent to weak pelvic floor muscle contraction was associated with reduced risk of bulge sensation after surgery.

## Introduction

Pelvic organ prolapse (POP) is a common condition that has a substantial impact on women’s quality of life, and the economic burden on society is significant [1]. The lifetime risk for undergoing POP surgery is 10–30%, and the recurrence rate after primary surgery is around 30–40% [2]. Known risk factors for recurrence after surgery are increasing age, high body mass index (BMI), higher parity, vaginal delivery, advanced stage of POP and family history [,^2^–^4^]. Levator ani muscle (LAM) trauma is associated with the development of POP and also POP recurrence in some studies [2]. One effect of LAM trauma is pathologically increased levator hiatal area (ballooning), which in turn causes a larger opening for possible descent of the pelvic organs [5–8]. These anatomical factors have been given great explanatory value in the understanding of POP development and recurrence after surgery, but functional factors have been less frequently investigated.

Levator ani muscle trauma is also associated with reduced ability to contract the pelvic floor [9]. Absent or weak pelvic floor muscle contraction has been suggested to be a risk factor for recurrence after primary correction of POP [7, 10], but this has been disputed in other studies, and the correlation between weak contraction and recurrence is low [11]. Most previous studies are retrospective, and the association between pelvic floor muscle contraction and anatomical and symptomatic failure has not been evaluated in a prospective setting. As the recurrence rates after POP surgery are high, there is a need to identify anatomical and functional risk factors for recurrence, to gain knowledge on how to prevent recurrence after prolapse surgery.

Our aim was to explore the impact of LAM trauma and pelvic floor contraction on symptoms and anatomy 6 months after POP surgery.

## Materials and methods

This study was a prospective, longitudinal cohort study of 200 women with POP scheduled for surgery at St. Olavs Hospital, Trondheim, Norway. It is a secondary analysis of a randomised controlled trial (RCT) on the effect of pelvic floor muscle training as a supplement to surgery (clinicaltrials.gov, NTC0364750) [12].

**Fig. 1:**
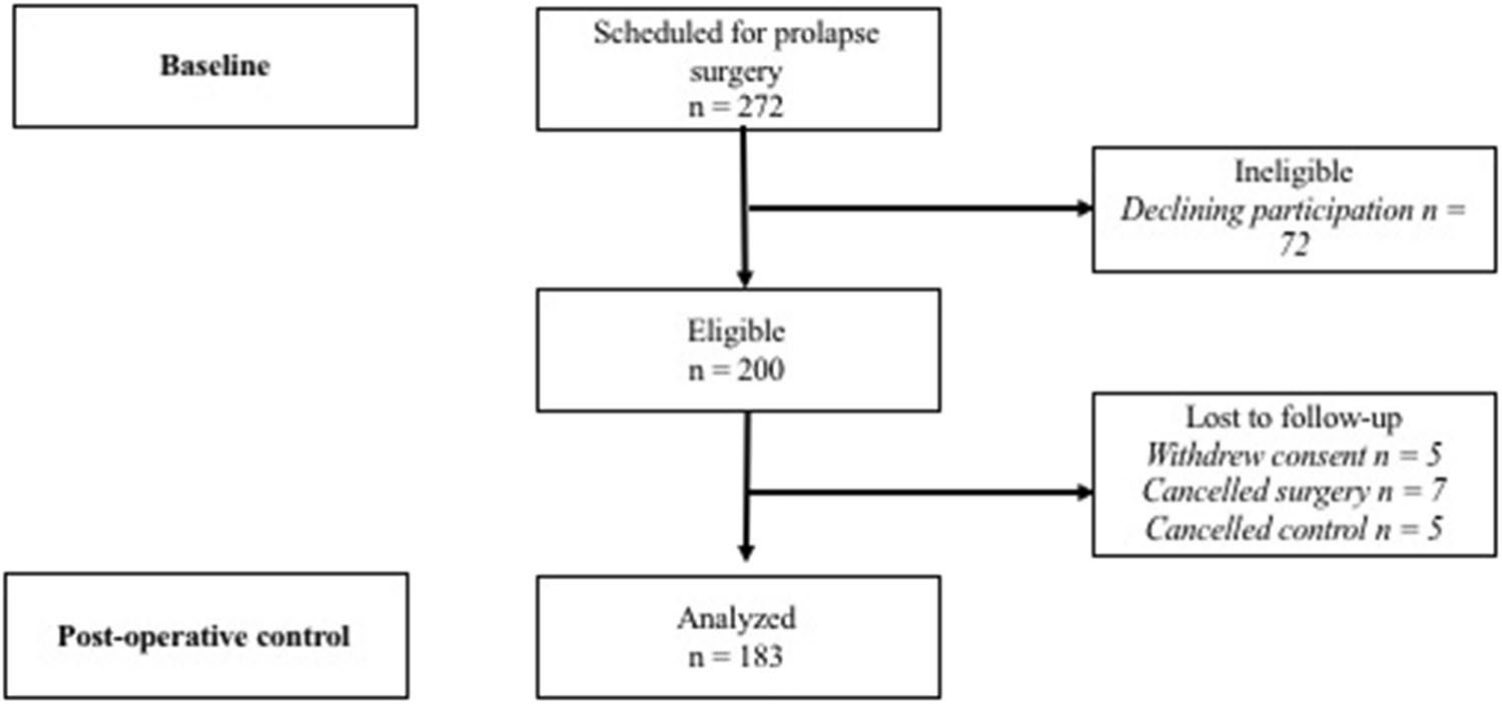
Flowchart of participants

Women were identified in the outpatient clinic between January 2017 and June 2018. Inclusion criteria were indication for POP surgery (POP stage ≥ 2 and bulge sensation), age over 18 years, ability to consent and understanding Norwegian or English language. They signed an informed consent at the inclusion visit, and the study was approved by the regional ethical committee (REK Midt 2015/1751). In total, 159 women were randomised. Forty-one women declined randomisation, but were included in a study on interrater reliability of transperineal ultrasound (Fig. 1) [13]. In the RCT we found no effect of intensive pelvic floor exercise on contraction, anatomy or symptoms after surgery, and therefore we combined the entire group in a cohort to study POP recurrence.

Women were examined by one of three examiners (MØN/SM/IV) at inclusion (approximately 3 months prior to surgery) and 6 months after surgery. They were seen for examination after bladder and bowel emptying and were thoroughly instructed in how to perform pelvic floor contraction and proper Valsalva [14]. POP stage was evaluated using the International Continence Society Pelvic Organ Prolapse Quantification (POP-Q) score [15]. POP-Q stage ≥ 2 was regarded as clinically significant. Women were asked whether or not they experienced a sensation of vaginal bulge [16]. Primary outcomes were anatomical POP-Q stage ≥ 2 (regardless of compartment) and sensation of bulge after surgery. A composite outcome was constructed combining POP-Q stage ≥ 2 and bulge sensation.

**Fig. 2:**
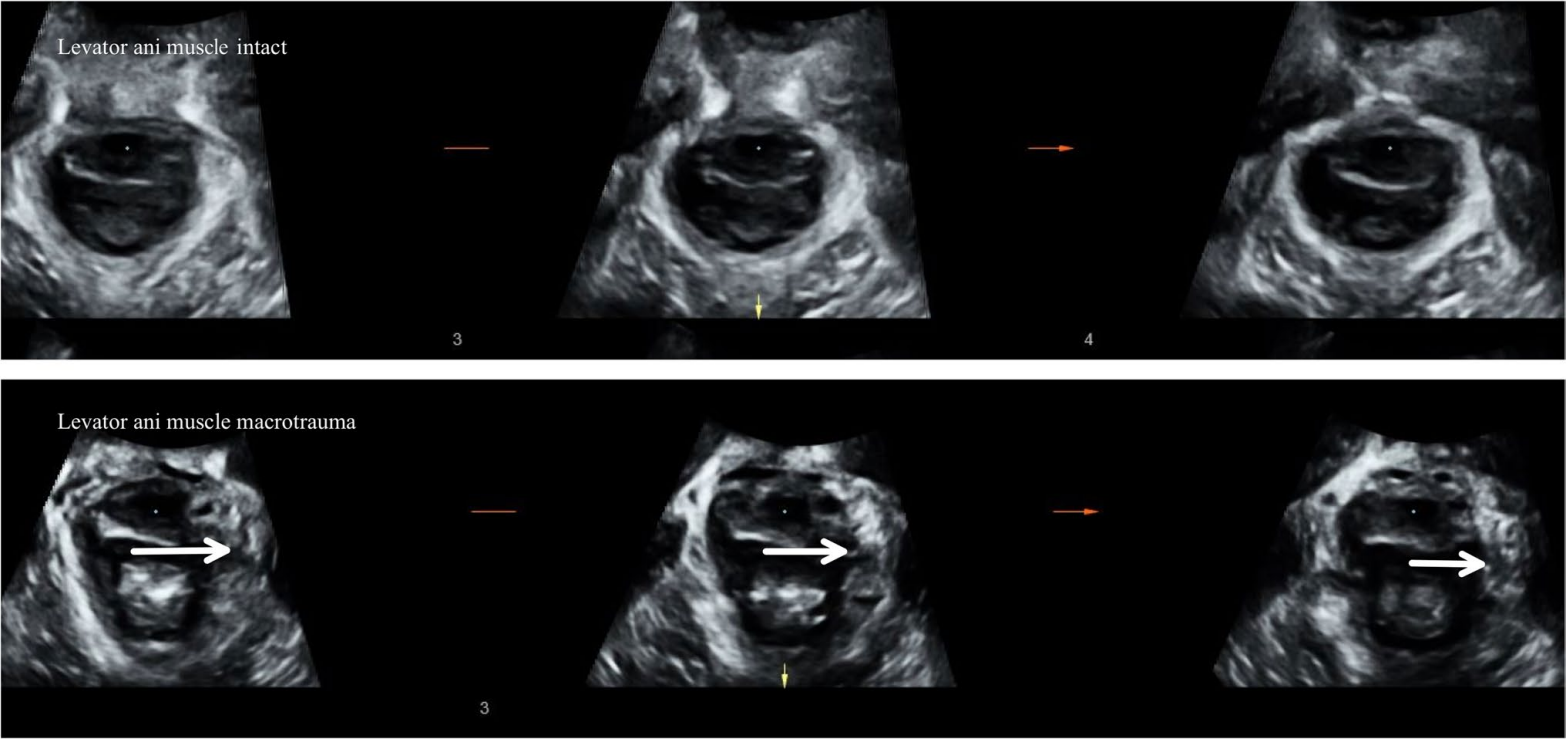
Levator ani muscle. Intact levator ani muscle (*top* illustrated by tomographic ultrasound imaging at three central slices (the plane of minimal hiatal dimensions, and 2.5 and 5.0 mm above). Unilateral levator ani trauma in the three central slices. The avulsion is illustrated by the *white arrow* on each image

**Fig. 3:**
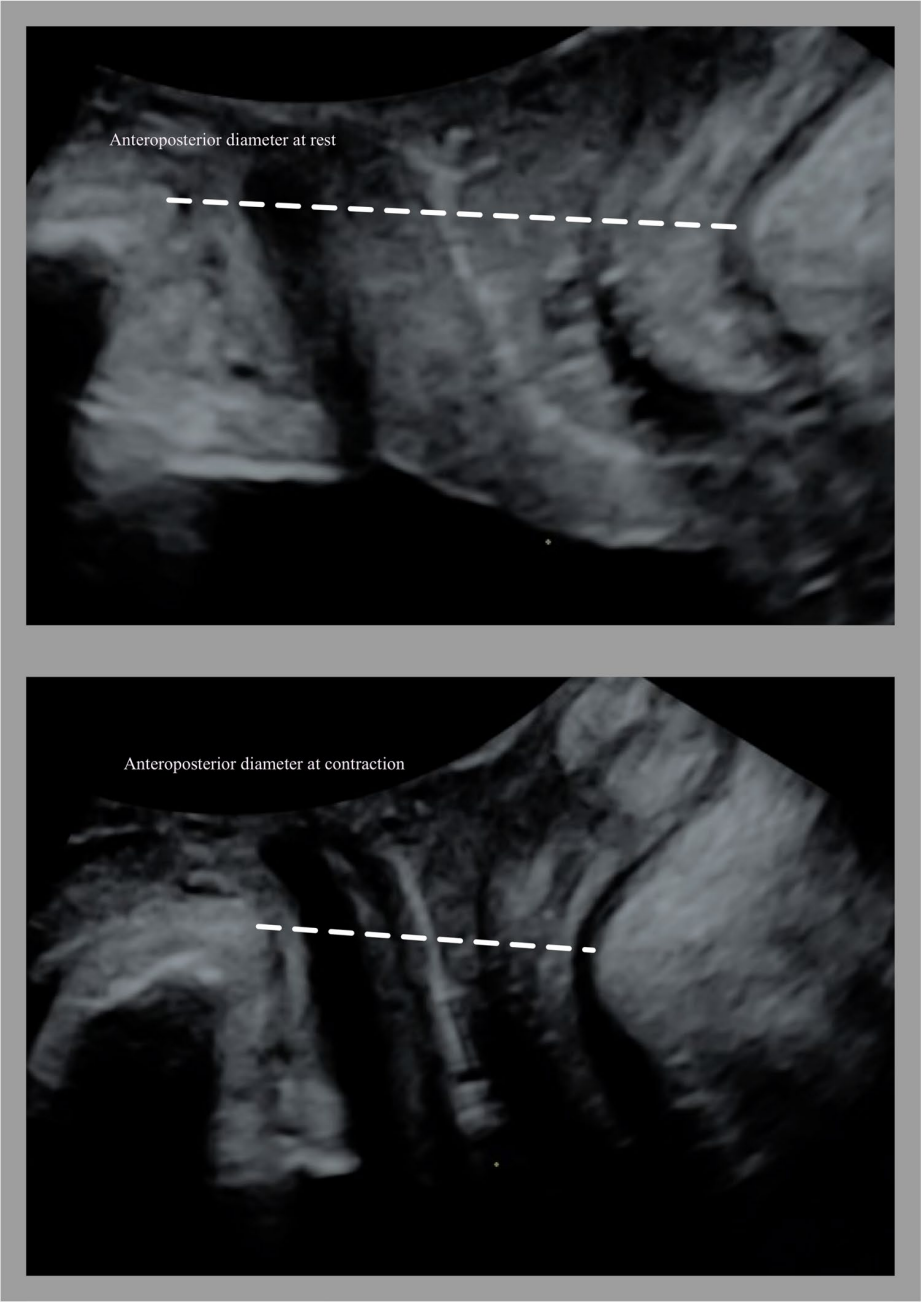
Assessment of pelvic floor contraction by ultrasound. Measurement of the anteroposterior diameter of the levator hiatus as the distance from the symphysis pubis to the puborectalis muscle at rest (*top*) and contraction (*bottom*)

Women were assessed using transperineal ultrasound. Three volumes were captured from rest to maximum contraction and three from rest to maximum Valsalva [17] using a Voluson GE S10 or E8 equipped with a RAB 4–8 MHz curved array transducer. The acquisition angle was set at 85° and the probe placed in the midsagittal plane. The volumes were analysed off-line a minimum 6–12 months after recording with 4D view by one examiner (MØN), blinded to clinical information. The offline analysis included assessment of LAM trauma, defined as abnormal insertion of the levator muscle on one or both sides of the pubic bone in all three central slices on tomographic ultrasound at contraction (Fig. 2) [18]. Pelvic floor contraction was assessed measuring the proportional change in levator hiatal anteroposterior diameter in 2D from rest to contraction using the formula: 100 × [(measurement _rest_ − measurement _contraction_)/measurement _rest_] (Fig. 3) [13]. Muscle contraction was categorised as absent (<1%), weak (2–14%), normal (15–29%) or strong (>30%) [13], and we dichotomised contraction into absent to weak or normal to strong.

Statistical analysis was performed using Statistical Package for the Social Sciences (SPSS) version 25. Normality was tested using histogram and QQ plots. We used the independent sample *t* test and the Mann–Whitney *U* test to compare means between groups for continuous background variables and the Chi-squared test to compare categorical background variables.

We used multivariate logistic regression models to study possible associations between LAM trauma and contraction (absent to weak versus normal to strong) with POP-Q stage ≥ 2, a bulge sensation and the composite outcome (POP-Q stage ≥ 2 and a bulge sensation) after surgery. We performed sub-analysis for recurrence in the compartment undergoing surgery and for new POP in a different compartment. We adjusted for age, parity, BMI, previous POP surgery, POP stage under or over 3 at baseline and previous hysterectomy. The level of statistical significance was set at 5%.

## Results

One hundred and eighty-three out of 200 women (92%) completed the study (Fig. 1). Background variables are outlined in Table 1. Women with impaired contraction were older at surgery. Thirty-four women (19%) underwent isolated anterior colporrhaphies and 32 (18%) posterior colporrhaphies. We performed vaginal hysterectomy in 21 (12%) and sacrocolpopexy/uteropexy in 19 (10%) of the women. Vaginal mesh was used in 4 women (2%). A combination of procedures was performed in 88 (48%) women. Overall anatomical recurrence (POP ≥ 2) was found in 76 (42%) women of whom 56 (31%) experienced recurrence in the compartment undergoing surgery and 9 (5%) in a new compartment. Twelve women (7%) had persisting prolapse in a compartment not surgically corrected during the procedure. Bulge sensation after surgery was reported by 35 out of 179 (19%). Only 25 out of 74 (34%) of women with POP ≥ 2 reported symptoms after surgery.

**Table 1:** Background characteristics for women with intact levator ani muscle trauma and women with absent to weak or normal to strong pelvic floor contraction. Continuous variables are given with mean (SD) and categorical variables with *n* (%).

	Levator trauma			Pelvic floor contraction		
	No, *n* = 91	Yes, *n* = 92	*p*	Normal to strong, *n* = 101	Absent to weak, *n* = 75	*p*
Age	62.2 (10.6)	60.6 (12.0)	0.202	60.5 (10.9)	63.6 (11.7)	0.024
BMI	26.3 (4.2)	26.0 (3.9)	0.760	26.2 (4.1)	25.9 (3.9)	0.627
Parity	2.6 (0.9)	2.5 (0.8)	0.086	2.6 (0.9)	2.5 (0.8)	0.320
Previous POP surgery	11 (12.1%)	15 (16.3%)	0.414	11 (10.4%)	15 (19.7%)	0.075
Previous hysterectomy	9 (9.9%)	16 (17.4%)	0.140	14 (13.2 %)	11(14.5%)	0.807
POP ≥ 3	51 (56.0%)	60 (65.2%)	0.204	65 (61.3%)	46 (60.5%)	0.914

*BMI* body mass index, *POP* pelvic organ prolapse

Women with LAM trauma were at an increased risk of POP ≥ 2 after surgery (Table 2). The prevalence of prolapse symptoms (bulge sensation) was similar for women with LAM trauma and an intact levator. Pelvic floor muscle contraction was not significantly associated with POP ≥ 2 after surgery (Table 2). Women with absent to weak contraction had a reduced risk of bulge sensation after surgery (Table 2).

**Table 2:** Impact of levator ani muscle trauma, and pelvic floor contraction on outcome variables. Adjusted odds ratio (aOR) are given with a 95% confidence interval after adjusting for age, body mass index, parity, POP stage at baseline, previous POP surgery and previous hysterectomy

	Levator trauma		aOR	Pelvic floor contraction		aOR
	Yes, (*n* = 92)	No, (*n* = 91)		Absent to weak (*n* = 76)	Normal to strong (*n* = 106)	
Anatomy, *n* = 183						
Any POP ≥ 2	46	30	2.1 (1.1–4.1), *p* =0.022*	35	41	1.5 (0.8–2.9), *p* = 0.233‡‡
Recurrence in same compartment	33	23	1.7 (0.9–3.4), *p* = 0.127**	24	32	1.0 (0.5–2.0), *p* = 0.985§
POP in new compartment	7	2	4.0 (0.8–20.7), *p* = 0.094	6	3	3.1 (0.7–13.2), *p* = 0.134
Symptoms, *n* = 179						
Bulge sensation	19	16	1.1 (0.5–2.4), *p* = 0.809	9	26	0.4 (0.2–0.9), *p* = 0.031§§
Composite *n* = 179						
Bulge sensation + any POP ≥ 2	14	11	1.3 (0.5–3.1), *p* = 0.612†	6	19	0.4 (0.1–1.0), *p* = 0.058±

*POP* pelvic organ prolapse, *aOR* adjusted odds ratio

*Preoperative POP ≥ 3: aOR 2.4 (1.2–4.8), *p* = 0.010

**Preoperative POP ≥ 3: aOR 2.2 (1.04–4.66), *p* = 0.038

†Preoperative POP ≥ 3: aOR 3.1 (1.1–9.0), *p* = 0.039

‡‡Preoperative POP ≥ 3: aOR 2.6 (1.3–5.1), *p* = 0.005

§Preoperative POP ≥ 3: aOR 2.3 (1.1–4.8), *p* = 0.030; age: aOR 1.05 (1.01–1.09), *p* = 0.009

§§Previous POP surgery: aOR 3.7 (1.2–11.9), *p* = 0.028

±Preoperative POP ≥ 3: aOR 3.1 (1.1–8.9), *p* = 0.041

In the multivariate analysis we found that preoperative POP ≥ 3 was associated with recurrence (POP ≥ 2) after surgery (Table 2). We found no difference in the results for contraction when avulsion was entered as a confounder.

## Discussion

We found an increased risk of POP ≥ 2 after POP surgery in women with LAM trauma and reduced risk for symptoms after surgery for women with absent to weak pelvic floor contraction. Preoperative POP ≥ 3 was associated with an increased risk of POP ≥ 2 in any compartment after surgery.

Increased risk of POP ≥ 2 after surgery in women with LAM trauma is in line with previous studies on risk factors for recurrence after POP surgery. A meta-analysis by Friedman et al. found OR 2.76 (2.17–3.51) for levator avulsion as a predictor of recurrence, which corresponds well with our finding (OR 2.1 (1.1–4.1) [2]. Increased risk of prolapse in a new compartment for women with LAM trauma is similar to other studies on women after POP surgery [19, 20]. A study by Oversand et al. on outcomes after POP surgery in the anterior compartment, however, showed no association with LAM trauma [21]. This study differs from the present study because all women underwent a Manchester procedure (anterior colporrhaphy, cervical amputation and posterior colpoperineorrhaphy), whereas women in our study had different procedures and only 15% underwent surgery with a Manchester procedure. Our study was not powered for a sub-analysis on the Manchester procedure.

The term contraction is disputed in the academic community as it is often used as a synonym for strength or contractility. One problem with using absolute change in diameter between rest and contraction is that this does not take into account the reduced potential for change in women with a small hiatus at rest. By using proportional change in this study, differences in resting hiatal dimensions are accounted for. This measure correlates well with other methods used for assessment of pelvic floor contraction, such as palpation and vaginal manometry, and it is reliable [13, 22].

No impact of absent to weak pelvic floor contraction on anatomical outcome also coincides with a previous study of anatomical recurrence [11]. We found that absent to weak contraction was associated with reduced risk of reporting bulge sensation after surgery and of a composite outcome of anatomical failure and symptoms (borderline significant). The women with normal to strong contraction were younger than women with absent to weak contraction. Younger women with pelvic floor disorders are more likely to report symptoms [23]. Also, young women are usually more physically active, including heavy physical work, and this may provoke symptoms [24].

Trauma to the pudendal nerve may cause impairment of both motor and sensory innervation in the pelvis [25, 26]. This provides a possible explanation for why women with impaired pelvic floor contraction at the same time could have reduced sensibility and thereby higher thresholds for reporting a bulge sensation in our study, but this needs further investigation. A short follow-up (6 months) may influence the results, and symptomatic prolapse may manifest later after surgery [27]. The healing process may not be complete, and sensation may be temporary reduced after surgery [28]. Women with absent to weak contraction had more previous POP surgery (borderline significant), which may further reduce the sensibility.

Levator ani muscle trauma and absent to weak pelvic floor contraction seem to increase the risk of development of POP in a new compartment. However, the numbers were small with wide confidence intervals and further studies are needed to confirm this result.

One strength of this study was the use of validated methods to evaluate LAM trauma, anatomy and symptoms after POP surgery. Our diverse population makes the results generalisable to clinical practice. However, the construction of the cohort may introduce a selection bias as we combined women from a randomised controlled trial with women declining participation.

The definition of anatomical recurrence (POP stage ≥ 2) in this study can be debated, as a POP stage 2 in the anterior compartment is often asymptomatic, whereas a stage 1 in the mid compartment can be symptomatic [29].

Inclusion of both primary and recurrent POP and surgery in different compartments can represent limitations of our study because anterior and posterior POP may have different aetiologies. There was also diversity in the surgical procedures, and the study sample size made sub-analyses for different procedures and compartments impossible. All women were instructed on how to contract the pelvic floor correctly. Knowledge of pelvic floor anatomy and contraction may have made these women perform better than a general patient population.

This research adds knowledge about the impact of LAM trauma and contraction on anatomical and symptomatic surgical outcomes. We suggest that evaluation of pelvic floor muscle trauma and ability to contract should be performed as a part of a standard gynaecological examination. Identification of women with LAM trauma can contribute to early prevention of POP development through pelvic floor muscle training [30] and postpone surgery. In addition, assessment of LAM trauma and contraction prior to POP surgery can influence choice of procedure and follow-up after surgery. The impact of sensitivity and function in women with prolapse needs further evaluation.

In conclusion, we found an increased risk of overall anatomical POP ≥ 2 after surgery in women with LAM trauma, but not an increased risk of bulge sensation. Women with absent to weak contraction had a reduced risk of bulge sensation after surgery.
